# Illinois Veterinary Medical and Surgical Association

**Published:** 1900-03

**Authors:** W. A. Swain

**Affiliations:** Secretary


Secretary.

### ILLINOIS VETERINARY MEDICAL AND SURGICAL ASSOCIATION.

The eleventh annual meeting of this Association was held at the office of Dr. S. H. Swain, Decatur, Ill., January 10th and 11th. The first session was called to order at 1 P.M., with Dr. J. Osborne, of Nokomis, in the chair. The minutes of the previous meeting were read and approved, followed by the President's address, after which Dr. J. M. Reed, of Mattoon, read a most excellent paper on "Equine Obstetrics," leaving very little room for discussion.

A recess of twenty minutes was then taken in order to allow those present to witness the operation by Dr. S. H. Swain of firing for splint.

Dr. S. H. Swain, being next on the programme for a paper on "Navicular Arthritis," asked permission of the members to be allowed to substitute a report of a case of rabies in a horse, which was granted. Report proved very interesting and instructive to those present.

The meeting was then adjourned on motion until 7 P.M., when Dr. I. M. Luzader, of Nokomis, read a paper on "Tetanus," followed by a general discussion of this and preceding papers, after which, on motion, the session was adjourned until 8 A.M., January 11th.

*January* 11, 1900.

Meeting called to order promptly at 8 A.M. by President Osborne, and Dr. R. W. Brathwait, of Champaign, made a very interesting talk on "Enteritis," dwelling upon the various stages of the disease and their diagnosis, and giving his treatment for the same.

On motion, recess was now taken to give those present an opportunity to witness a post-mortem by Dr. A. Travis, of Litchfield, and Dr. R. W. Brathwait, of Champaign, on a cow which had died the preceding night of tuberculosis, and had been ordered brought to the sanitarium for that purpose.

Dr. V. G. Hunt, of Arcola, next read a paper on "Hydrophobia Equinine," responded to by Dr. R. W. Brathwait and Dr. S. H. Swain. This being a conclusion of the programme for the present meeting, the Association proceeded to hear the reports of the various committees, which were read and approved.

Officers were next elected for the ensuing year, as follows: President, Dr. V. G. Hunt, Arcola, Ill.; First Vice-President, Dr. J. H. Osborne, Nokomis, Ill.; Second Vice-President, Dr. J. M. Reed, Mattoon, Ill.; Secretary, Dr. W. A. Swain, Mt. Pulaski, Ill.; Treasurer, Dr. S. H. Swain, Decatur, Ill.

The newly-elected officers were then installed, and after the President had appointed the Standing Committees, the meeting was adjourned, on motion, to meet at Mattoon, Ill., August 8 and 9, 1900.

W. A. Swain,
Secretary.

